# Molecular Dynamics, Dielectric Properties, and Textures of Protonated and Selectively Deuterated 4′-Pentyl-4-biphenylcarbonitrile Liquid Crystal

**DOI:** 10.3390/ma17205106

**Published:** 2024-10-19

**Authors:** Jadwiga Tritt-Goc, Magdalena Knapkiewicz, Piotr Harmata, Jakub Herman, Michał Bielejewski

**Affiliations:** 1 Institute of Molecular Physics, Polish Academy of Sciences, M. Smoluchowskiego 17, 60-179 Poznan, Poland; jtg@ifmpan.poznan.pl (J.T.-G.); magdalena.knapkiewicz@ifmpan.poznan.pl (M.K.); 2 Institute of Chemistry, Military University of Technology, ul. gen. S. Kaliskiego 2, 00-908 Warsaw, Poland; piotr.harmata@wat.edu.pl (P.H.); jakub.herman@wat.edu.pl (J.H.)

**Keywords:** nematic liquid crystal, 5CB series, molecular dynamics, fast field cycling NMR relaxometry, deuteration procedure, LC textures

## Abstract

Using liquid crystals in near-infrared applications suffers from effects related to processes like parasitic absorption and high sensitivity to UV-light exposure. One way of managing these disadvantages is to use deuterated systems. The combined ^1^H and ^2^H nuclear magnetic resonance relaxometry method (FFC NMR), dielectric spectroscopy (DS), optical microscopy (POM), and differential scanning calorimetry (DSC) approach was applied to investigate the influence of selective deuteration on the molecular dynamics, thermal properties, self-organization, and electric-field responsiveness to a 4′-pentyl-4-biphenylcarbonitrile (5CB) liquid crystal. The NMR relaxation dispersion (NMRD) profiles were analyzed using theoretical models for the description of dynamics processes in different mesophases. Obtained optical textures of selectively deuterated 5CB showed the occurrence of the domain structure close to the I/N phase transition. The dielectric measurements showed a substantial difference in switching fields between fully protonated/deuterated 5CB and selectively deuterated molecules. The DSC thermograms showed a more complex phase transition sequence for partially deuterated 5CB with respect to fully protonated/deuterated molecules.

## 1. Introduction

Liquid crystals (LCs) are materials that combine order and mobility on a molecular and supramolecular level, displaying physical properties that are dependent on the self-organization and molecular dynamics of LC molecules. The versatile and unique properties of these materials and their mixtures are mostly associated with display technologies. However, the high sensitivity to external physical and chemical environments influencing the self-organization of LC molecules enables them to be used in a variety of other fields, such as LC-based chemical sensors [1], biosensors [2], photovoltaics [3] and nanophotonics [4], light-emitting diodes [5], molecular motors [6], field-effect transistors [7], and lasers [8,9,10]. The possibility of obtaining bi- and multicomponent mixtures of LC materials and chemical modification of base materials allows for obtaining LC metamaterials with a broad range of modifications, e.g., magnetic or chromonic properties [11].

Although liquid crystals have been known since the late 19th century [12] and since then have been intensively studied and developed, there are still some aspects where improvement needs to be carried out to utilize liquid crystals in new fields. One such emerging area is near-infrared range applications where mesomorphic organic materials exhibit several disadvantages, such as parasitic absorption. This unwanted effect relies on the presence of absorption from molecular vibration bands, their overtones, and combination bands, primarily related to the vibrations of C-H bonds in rings and alkyl chains [13,14,15]. LCs used in devices operating in the infrared are commonly modified, as an example, we can distinguish dye-doped chiral nematic liquid crystals [16] or polymer-dispersed liquid crystal/graphene oxide nanocomposites [17].

A natural way to reduce absorption in this electromagnetic spectrum range is to treat the molecule as a harmonic oscillator and increase the mass of one of the oscillator’s elements, specifically the C-H bond. This adjustment reduces the frequency of its fundamental vibrations and shifts the absorption bands toward longer wavelengths. One of the possible alternatives that can be considered for the C-H bond is to replace the C-H bond with a C-D bond. The fundamental valence vibration frequency of the C-H bond (3030–2778 cm^−1^, or 3.3–3.6 μm) is shifted to the range of 2272–2040 cm^−1^ (4.4–4.9 μm) for C-D bonds. This shift also affects the first harmonic of the C(aryl)-H bond deformation vibration, moving it from approximately 5882 cm^−1^ (1.7 μm for C-H) to 4348 cm^−1^ (2.3 μm for C-D). The higher harmonics are of minimal importance due to their very low intensity, less than 0.001 times the base intensity.

The idea of using deuterated materials for this purpose has been known since the 1980s [15,18]. However, the complexity of synthesizing deuterated mesogens has effectively prevented a more comprehensive range of materials from being obtained and studied. There are reports in the literature on the synthesis of deuterated liquid crystals, but all are limited to the presentation of the main homolog of the thermotropic liquid crystal family, i.e., 4-cyano-4′-pentylbiphenyl 5CB (also used abbreviated name PCB) [18,19,20,21].

Deuterated 5CB liquid crystal materials can be synthesized using two primary methodologies: the hydrogen–deuterium exchange method and direct synthesis from deuterated precursors. The hydrogen–deuterium exchange method involves creating the conventional hydrocarbon skeleton, specifically 4-alkylbiphenyl, which undergoes hydrogen–deuterium exchange using catalysts like palladium or platinum and D_2_O as the deuterium source, with the polar CN group introduced at the final step. However, this method often results in incomplete deuterium exchange, especially in the alkyl chain segments, achieving less than 85–90% deuteration. Alternatively, the direct synthesis method starts with deuterated raw chemicals of high purity (over 99% deuterium atoms) and synthesizes the liquid crystal molecule through conventional multistep organic synthesis, ensuring a high level of isotopic purity throughout the molecule. The materials discussed here were synthesized following our modified literature procedure described in ref. [20].

Additionally, to reduce parasitic absorption in the NIR spectral range in deuterated LCs, another promising aspect of substituting the ^1^H atoms in the structure of the molecules with the ^2^H isotope has been found. Our latest work on deuterated-labeled liquid crystal materials has shown that such materials exhibit enhanced stability under ultraviolet (UV) radiation compared to their nondeuterated counterparts [22,23]. Compared to hydrogen, the increased mass of deuterium atoms results in stronger C-D bonds that are less prone to photochemical degradation. This enhancement in bond strength reduces the rate of bond cleavage and the formation of reactive intermediates under UV exposure, thereby improving the overall durability and longevity of liquid crystal materials when subjected to UV light. This discovery opens new possibilities for applying deuterated liquid crystals in environments with high UV exposure, extending their functional lifespan and performance. However, to successfully utilize deuterated liquid crystals for applications in a wider spectral range of the electromagnetic field, the influence of selective and controlled isotopic substitution on crucial physical properties must be determined.

In the current study, we chose the 4′-pentyl-4-biphenylcarbonitrile (5CB) liquid crystal as a model system to investigate the influence of selective deuteration on its molecular dynamics, mesophase occurrence, and electrical properties. The 5CB is a representative of widely used material whose properties have been investigated by different experimental techniques, allowing us to determine its properties accurately. However, most experimental techniques deliver only a single and averaged component of some second-rank property like susceptibility, permittivity, or refractive index. Because most of the mesogenic materials stay in dynamic equilibrium at a given LC phase, they involve a number of conformational states that average over the molecular orientations, with intra- and intermolecular interactions fluctuating in time. To investigate the molecular dynamics and its dependence on the self-organization of LC molecules, we used the nuclear magnetic resonance relaxometry method in the function of the external magnetic field to determine the spectral density functions of the investigated systems. To confirm and check how the chemical structure of the LC molecule affects its physical properties, we selectively deuterated 5CB, introducing deuterons either in the core of the molecule (5CB-*d*_8_) or substituting all hydrogen atoms (5CB-*d*_19_) and compared the results with the fully protonated 5CB molecule.

The purpose of the current work is to study the influence of controlled deuterization on spin–lattice relaxation mechanisms, phase occurrence, and thermal properties in 5CB liquid crystal in isotropic and nematic phases to assess the information on the effect of deuterization degree on molecular dynamics and self-organization of LC molecules chemically modified for utilization in near-infrared applications. Our aim is to determine if observed differences in nematic phase textures, the presence of additional phase transition peaks, and responsiveness to external electric fields observed for different degrees of deuteration can be related to local dynamic processes. The advantage of the NMR FFC method is the accessible time scale of motions that can be detected in the range from 10^−8^ to 10^−3^ s, being specifically sensitive for slow motions that are pronounced for different mesophases in the low-frequency range.

## 2. Materials and Methods

The samples of 5CB, 5CB-*d*_8_, and 5CB-*d*_19_ were synthesized in the LC group of the Military University of Technology, Warsaw, according to the procedure described elsewhere [20]. Table 1 presents the structural formulas of the studied materials.

### 2.1. NMR Relaxometry

The molecular dynamics investigations were performed using the nuclear magnetic resonance method and fast field cycling relaxometry technique. The NMR dispersion (NMRD) profiles were recorded on a SpinMaster2000 relaxometer from Stelar Company, Mede, Italy. Measurements of spin–lattice relaxation rates were performed as a function of external magnetic field B_0_ strength expressed in Larmor resonance frequency units for protons in the range from 10 kHz to 20 MHz and as a function of temperature. The dependence of the relaxation rate (R_1_ = 1/T_1_) on Larmor frequency is called the dispersion profile and abbreviated as NMRD. The NMRD profiles provide information on motions occurring on time scales from 10^−4^ to 10^−9^ s. Its shape reflects the spectral density function *J*(ω), which is a Fourier transform of the correlation function G(t). Time-correlation functions formally describe translational and reorientational motions in physical terms [24]. In the simplest case, the NMR relaxation phenomenon is driven by the stochastic fluctuations of the local magnetic fields and magnetic interactions between the spins, e.g., dipole–dipole interactions. These fluctuations are characterized by a specific time constant called correlation time. The correlation time describes the time scale of losing the information by the molecule (spins) on its initial state. The cause of the fluctuations that determine the relaxation mechanisms are molecular dynamical processes that the molecules undergo [25,26,27]. In the case of only one dynamical process characterized by a single value of correlation time (referred to as the Bloembergen–Purcell–Pound (BPP) model), the spectral density function can be described as follows [28,29]:

$$J\left(\omega \right)=\frac{\tau }{1+{\;\omega }^{2}\tau^{2}}$$

where *τ* is the characteristic time of the fluctuations, the correlation time, and ω is the Larmor frequency. By analyzing the shape of the spectral density function *J*(ω), we obtain access to the relaxation mechanism, allowing us to probe the molecular dynamical processes of investigated systems. The *J*(ω) function reaches its maximum value when the characteristic time of the local fluctuations equals the reciprocal of the Larmor frequency $\tau_{c}=\omega^{-1}$.

Depending on the nature of the dynamical processes responsible for the relaxation mechanisms, different theoretical models for analyzing the spectral density functions have been developed (see Section 3.5). For the same molecular motion, the correlation time may differ depending on the temperature, following, e.g., the Arrhenius law, causing the spectral density function to extend toward higher frequencies with decaying amplitude. With this assumption, we can treat the spectral density function as a measure of the probability of spin transitions of a frequency *ω* induced by the fluctuation of local magnetic fields characterized by the given correlation time *τ_c_*. More macroscopically speaking, this shows that molecular motion described by a time scale of *τ* = *ω*^−1^ is the most efficient source of relaxation at a given frequency (and thermal conditions) [30].

The advantage of the field-cycling NMR relaxometry method lies in the access to a low-frequency range. Most information on slow dynamical processes characterized with correlation times longer than nanoseconds is reflected in the spectral density at low values of *ω*, corresponding to magnetic fields below 1T, much below high-resolution NMR spectrometers. Therefore, this method is very useful in studying, e.g., slow collective motions that depend on molecular size and shape, viscosity, temperature, and intermolecular interaction. These features of the NMR FFC technique make it ideal for investigating the molecular dynamics of LC molecules under various mesophases occurring as a function of temperature. The technical details of the FFC NMR relaxometry experiment are reported elsewhere [31,32].

### 2.2. Thermal Properties and Phase Transition Measurements

The mesophase sequence and phase transition temperature were measured using the differential scanning calorimetry method (DSC) and a Perkin-Elmer DSC4000 instrument (Perkin-Elmer, Waltham, MA, USA). The measurements were performed under a gaseous nitrogen atmosphere in the temperature range from 273 K to 333 K, covering transitions from isotropic to crystal phase. The LC samples were placed in a sealed aluminum crucible and subjected to three consecutive heating and cooling cycles at a scanning rate of 5 °C/min.

### 2.3. Mesophase Texture Analysis

The mesophase texture images were recorded with an Olympus optical microscope BX53-P (Tokyo, Japan) under a magnification of 200× and crossed polarizers. The samples were placed on a glass microscopic slide and covered with a 0.2 mm cover slip. The sample thickness was 20 μm and the temperature of the sample was stabilized and controlled with a Linkam TSM91 hot stage. The images were taken during the heating cycle.

### 2.4. Dielectric Spectroscopy Measurements

The dielectric spectroscopy measurements were performed for protonated and deuterated samples with the use of an HP 4192A LF Impedance Analyzer (Hewlett-Packard, Tokyo, Japan) connected with a Mettler FP–82 HT hot stage, at a frequency of 1.1 kHz, oscillation level at 0.1 V, and different BIAS voltages at 5, 10, 15, and 20 Celsius degrees below the I/N phase transition.

## 3. Results and Discussion

### 3.1. NMR Spectroscopy Investigations

**Figure 1:**
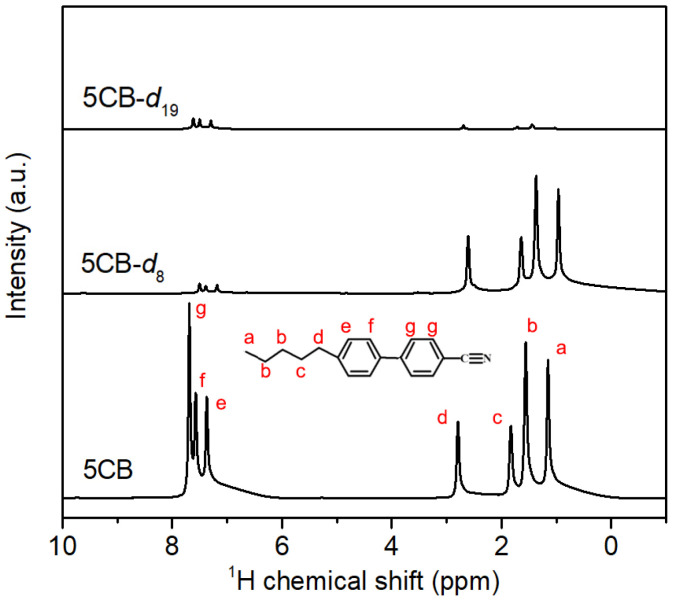
The ^1^H NMR spectra of 5CB, 5CB-*d*_8_, and 5CB-*d*_19_ in the isotropic phase.

The selectivity and efficiency of used synthesis routes to prepare partially and fully deuterated 5CB samples were determined by proton NMR spectroscopy studies. The NMR spectrum of the fully protonated 5CB sample was used as a reference. The same volume of all studied LC samples was placed in 5 mm NMR tubes and subjected to measurements at 50 °C, resulting in an isotropic phase. Figure 1 presents recorded spectra for 5CB, 5CB-*d*_8_, and 5CB-*d*_19_ samples measured under the same conditions without scaling of the spectra. Due to the high sensitivity of the ^1^H nucleus, even a very small quantity of hydrogen atoms left after deuteration can be easily detected. Based on integral analysis of the ^1^H spectra, we estimated the degree of conversion for partially and fully deuterated samples and the selectivity of this process. For the 5CB-*d*_19_ sample, the total deuteration degree was equal to 96%, where the hydrogen atoms in the benzene rings were substituted with deuterons in 93% and the alkyl chain in 97%. In the case of the 5CB-*d*_8_ sample, the total deuteration degree was equal to 47%, where the substitution degree in the aromatic part was equal to 96% and in the side chain to 11%—the result obtained proves that a very high degree of conversion was acquired using the synthesis route. Also, the substitution process selectivity remained at a high level, allowing us to consider the 5CB-*d*_8_ sample as selectively deuterated.

### 3.2. Thermal Properties and Phase Transitions—DSC

**Figure 2:**
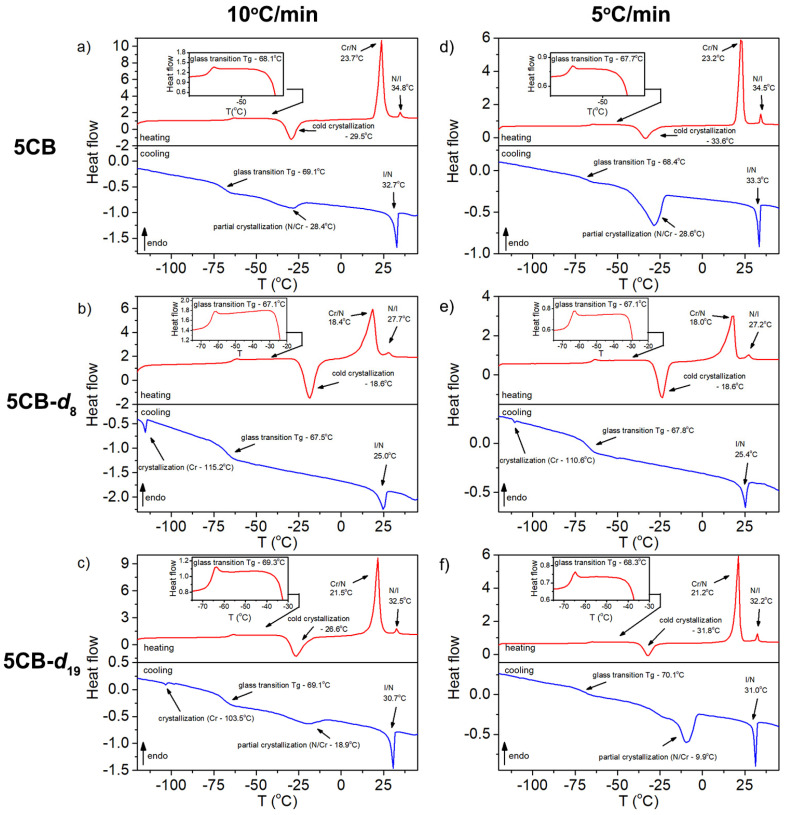
DSC heating and cooling curves for 5CB (**a**), 5CB-*d*_8_ (**b**), and 5CB-*d*_19_ (**c**) at 10 °C/min and for 5CB (**d**), 5CB-*d*_8_ (**e**), and 5CB-*d*_19_ (**f**) at 5 °C/min heating rate.

The thermal properties and phase transition temperatures of 5CB liquid crystal, especially the temperature of the transition from isotropic to nematic phase, are well known in the literature and have been previously determined [33]. In this report, we investigated the thermal properties of 5CB in a broad temperature range and accounted for the transitions from isotropic to crystal phase in order to characterize it as a reference for the same thermal treatment of selectively deuterated 5CB samples. Figure 2 presents the recorded DSC curves for the heating and cooling cycles of the investigated samples. The results clearly show that the degree of deuteration influences the phase transition temperatures and the presence of individual exothermic features. Some similarities can be found for fully protonated and fully deuterated 5CB molecules, whereas for selectively deuterated 5CB-*d*_8_ molecules, the differences in characteristic phase transition temperature are more pronounced; additionally, the partial crystallization observed for 5CB and 5CB-*d*_19_ during cooling stage is missing. In a fully protonated 5CB molecule (Figure 2a), we can observe endothermic peaks related to crystal-to-nematic (Cr/N) and nematic-to-isotropic (N/I) phase transitions, a glass transition (Tg) feature, and a cold crystallization exothermic peak during heating of the sample. The temperature determined for individual features agrees well with data found in the literature [33]. The most dominant peak observed at 23.7 °C is related to the Cr/N phase transition, followed by the N/I phase transition at 34.8 °C. Upon heating the sample from −120 °C, the transition from glassy to liquid phase at −68.1 °C (Tg—fictive temperature) followed by cold crystallization (exothermic peak at −29.5 °C) was observed. It is worth noticing that the thermal history of the sample affects observed features, e.g., if the sample is cooled after completion of cold crystallization and measured upon heating in the region where the glass transition was previously observed—no glass transition feature is present, proving that the sample was completely crystallized. Therefore, the endothermic peak observed after cold crystallization can be determined as a Cr/N phase transition. During the cooling of the 5CB sample first observed, a sharp exothermic peak at 32.7 °C is related to the transition from an isotropic to a nematic phase (I/N) followed by a broad exothermic peak at −28.5 °C caused by partial crystallization. Subsequent cooling of the sample indicates a glass transition feature related to the transition from the nematic liquid-to-nematic glassy state at −69.1 °C. In the 50 °C to −120 °C temperature range with heating and cooling of the sample at 10 °C/min, no crystallization peak after glass transition in the cooling stage of the 5CB sample was observed in any of the three subsequent heating–cooling cycles.

In the case of the fully deuterated 5CB-*d*_19_ sample (Figure 2c), the same sequence of endo- and exothermic peaks in heating and cooling cycles with only small differences in the temperatures of particular transitions to the corresponding transitions in the protonated 5CB sample were observed. One additional feature was observed for the 5CB-*d*_19_ sample during cooling at 10 °C/min, which was detected after the glass transition as a small exothermic peak at −103 °C related to the complete crystallization of the sample. This peak was confirmed in three subsequent cooling–heating cycles in the investigated sample. To explain the same phase transition behavior of the fully protonated 5CB and fully deuterated 5CB-*d*_19_ samples, we need to consider the fact that complete deuteration of the sample does not introduce any change considering the symmetry and imbalance of the intermolecular interactions between the molecules, which is the case for the partially deuterated 5CB sample.

The most interesting case is observed for a partially deuterated 5CB-*d*_8_ molecule (Figure 2b). In this case, the ^2^H atoms substituted only ^1^H atoms in the aromatic core of the molecule, leaving the alkyl chain unchanged. In this case, the mass distribution in the molecule changes, and now, we have a heavy organic core and light alkyl chain. Additionally, the intermolecular interactions are also changed as deuterons have approx. 2.5 times larger size, 3.2 smaller magnetic dipole moment, 2 times larger mass, and same electric charge, but are distributed over a larger volume compared to protons. The first noticeable change with respect to 5CB and 5CB-*d*_19_ is the greater width of the observed exothermic and endothermic peaks. Next, during the cooling of the 5CB-*d*_8_ sample, no partial crystallization was observed before the glass transition at −67.5 °C. Subsequent cooling leads to a sharp and well-pronounced exothermic peak related to crystallization at −115.2 °C. Upon heating the sample, the analogous sequence of exothermic and endothermic features was observed as in the 5CB and 5CB-*d*_19_ samples with greater differences in individual phase transition values. Table 2 gathers all the characteristic phase transition temperatures for all the investigated samples measured with the DSC and POM methods.

All samples were measured for two temperature change rates, 10 °C/min and 5 °C/min. With the decrease in the heating/cooling rate, the temperatures for individual phase transitions showed only small differences except for cold crystallization in fully protonated and fully deuterated samples and partial crystallization in fully deuterated samples, where the phase transition temperature changes were more pronounced. This interesting effect is worth future investigation with more heating/cooling change rates and isothermal measurements.

In summary, when the 5CB is cooled at 10 °C/min, the sample is in the isotropic phase at temperatures above 33 °C, in the nematic phase in the temperature range from −25 °C to 33 °C, and in supercooled nematic liquid with a small fraction of crystallites below −25 °C. Upon heating, the 5CB behaves as supercooled nematic liquid up to −30 °C, in the temperature range from −30 °C to 24 °C it is in crystalline form, in the nematic phase in the range from 24 °C to 35 °C, and finally in isotropic phase above 35 °C.

For the 5CB-*d*_8_ cooled at 10 °C/min, the sample exists in an isotropic phase above 25 °C, in the nematic/supercooled nematic phase for temperatures between −60 °C and 25 °C, and in glassy nematic liquid in the temperature range from −115 °C to −60 °C, with crystallization at −115 °C. Upon heating, the sample behaves as supercooled nematic liquid up to −19 °C and as crystalline in the temperature range between −19 °C and 19 °C. It exists in the nematic phase between 19 °C and 28 °C and in the isotropic phase above 28 °C.

The 5CB-*d*_19_ during cooling at 10 °C/min exists in the isotropic phase above 31 °C, in the nematic phase in the temperature range from −19 °C to 31 °C, and as the supercooled nematic liquid with a fraction of crystallites below −19 °C, with crystallization at −103 °C. During heating, the 5CB-*d*_19_ behaves as supercooled nematic liquid up to −27°C, as crystalline between −27 °C and 21 °C, as nematic in the temperature range from 21 °C to 32.5 °C, and as an isotopic phase above 33 °C.

### 3.3. Mesophase Texture Analysis

**Figure 3:**
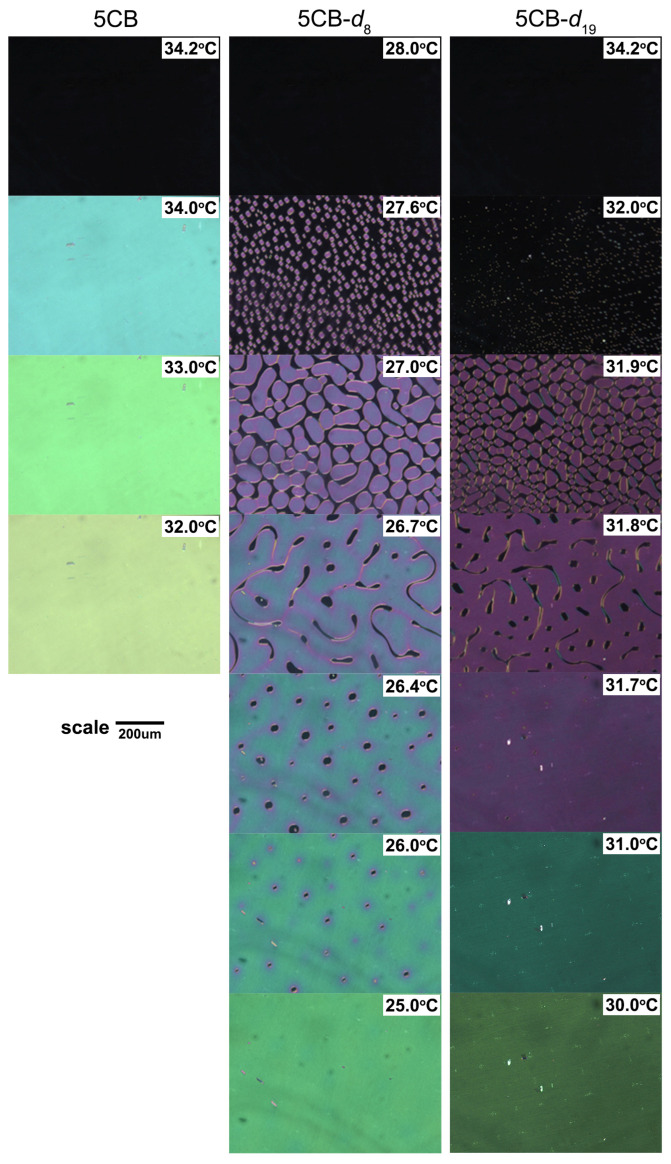
Polarization microscopy images of 5CB, 5CB-*d*_8_, and 5CB-*d*_19_ liquid crystal.

To investigate the influence of deuteration on the intermolecular interactions and self-organization of the 5CB, 5CB-*d*_8_, and 5CB-*d*_19_ molecules, we recorded and compared the LC textures in the temperature range of the nematic phase existence. Substantial differences between protonated and all deuterated samples were observed. All recorded textures are presented in Figure 3. In the case of the protonated 5CB sample on reaching the temperature of nematic phase existence, the texture’s uniform color was observed, confirming the homogeneous alignment of the LC molecules throughout the cell. Further, decreasing the temperature reduced the thermal vibrations and led to an increase in the ordering parameter, which is reflected by the change in the texture color. In the case of the 5CB-*d*_8_ sample, the domain texture was observed upon reaching the transition from isotropic to the nematic phase (I/N). At proximity to the I/N phase transition, small and separated regions of the nematic phase were homogeneously spread in the continuous isotropic phase. Further decrease in the temperature caused the growth of the nematic domains, which gradually started to be in contact with its boundaries. At this stage, the volume of the isotropic phase was decreased but remained continuous. Further decrease in temperature led to merging of the individual nematic domains and vanishing of the domain walls. The trace of vanishing domain walls is seen as a gradual change in the texture’s color in merged domains. For sufficiently large domains, the same gradient of the texture color is observed close to domain walls. This effect indicates that the alignment of the 5CB-*d*_8_ molecules is different at domain walls and in the center of the domain. Further decreases in temperature lead to the merging of all nematic domains, making the isotropic phase become single and separated domains that finally disappear. After the disappearance of the domains of the isotropic phase, the color of the texture of the nematic phase becomes uniform throughout the whole cell. This effect indicates that the alignment of the 5CB-*d*_8_ molecules becomes uniform in the entire volume. In the case of fully deuterated 5CB-*d*_19_, the domain texture of nematic regions occurred at the onset of the I/N phase transition. Further decrease in temperature leads to the growth of the nematic domains, which eventually merge. During the development of the nematic phase domains, the isotropic regions follow the behavior observed in 5CB-*d*_8_. However, in the case of 5CB-*d*_19_ molecules, no gradient of the texture color in nematic domains was observed even after merging. Therefore, no trace of the vanishing domain walls was observed. This indicates that alignment in the nematic domains of 5CB-*d*_19_ molecules remains uniform and homogenous, close and far from the domain walls. A change in the texture color for 5CB-*d*_19_ was observed upon cooling after the domains of the isotropic phase had vanished, meaning that 5CB-*d*_19_ follows the order parameter behavior as a function of temperature as in other studied 5CB samples. It is worth noticing that the whole process of occurrence and growth of the nematic phase was completed in a much narrower temperature range than for 5CB-*d*_8_.

### 3.4. Dielectric Spectroscopy Measurements

**Figure 4:**
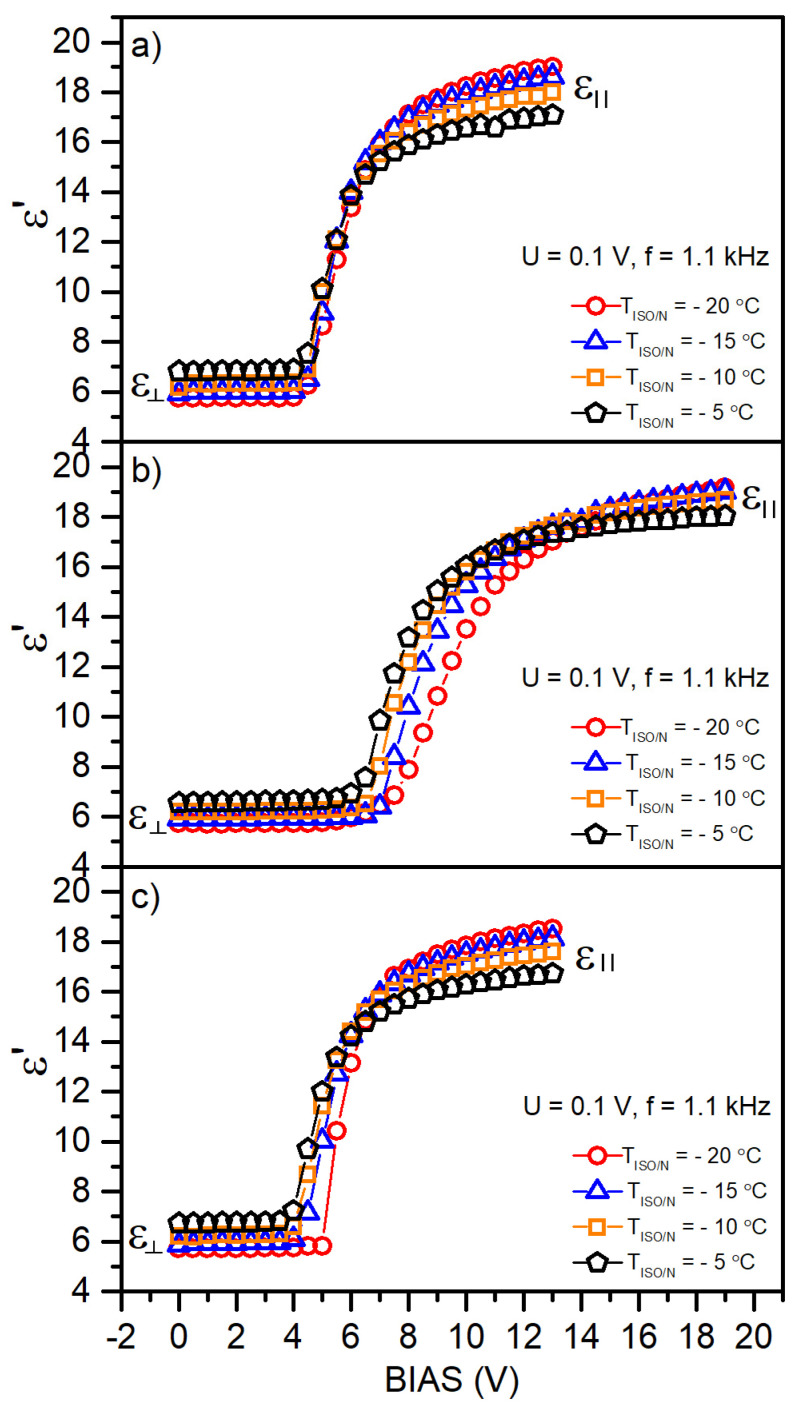
Electrical permittivity as a function of the voltage of the BIAS external field for the nematic phase in the studied materials: (**a**) 5CB, (**b**) 5CB-*d*_8_, and (**c**) 5CB-*d*_19_.

**Figure 5:**
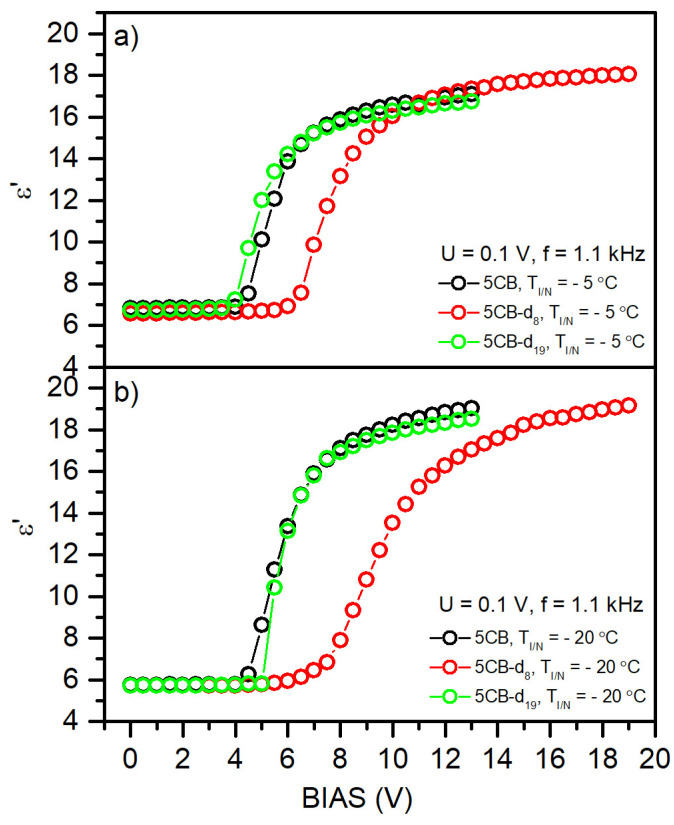
Electrical permittivity *ε* dependence upon the BIAS electric field for the 5CB, 5CB-*d*_8_, and 5CB-*d*_19_ materials at reduced temperature T_I/N_ −5 °C (**a**) and −20 °C (**b**).

In dielectric liquid crystals, by applying an external electric field, it is possible to reorient the LC molecules according to the direction of the applied field. In nematic liquid crystals, the direction of the optical axis is described with the director ***n***, representing the average unit vector along the molecular axis. By changing the director’s orientation, the optical properties are also changed. In the absence of the external electric field, the orientation of *n* is determined by the external interactions and anchoring effect. Applying the electric field, which is initially perpendicular to the director, causes the deformation of a homogenous layer of LC molecules above a specific strength of the electric field, called the threshold voltage. For sufficiently high fields, it is possible to completely reorient the director from a perpendicular to parallel orientation. This effect is possible due to the anisotropy of the dielectric permittivity $∆\varepsilon =\varepsilon_{∥}-\varepsilon_{⊥}$, where $\varepsilon_{⊥}$ and $\varepsilon_{∥}$ is the electric permittivity measured along the directions perpendicular and parallel to *n*, respectively. With positive dielectric anisotropy (Δ*ε* > 0), the director is forced to align along the electric field. To investigate the influence of selective deuteration on the dielectric properties of studied 5CB derivatives and interactions between the studied molecules, we analyzed the dependence of the electric permittivity *ε*′ on the BIAS electric field. Figure 4 shows recorded profiles for the 5CB, 5CB-*d*_8_, and 5CB-*d*_19_ samples at different temperatures within the nematic phase. Based on the results, we can conclude that partial deuteration influences the dielectric permittivity and threshold voltage of investigated samples the most. The calculated parameters are gathered in Table 3. The possible source of this effect is related to changes in charge distribution over the 5CB-*d*_8_ molecules and D/H intermolecular interactions, which are stronger than H/H interactions. Moreover, the DSC investigations revealed additional phase transitions within the nematic phase, which can induce more complex interaction with the external electric field due to different molecule alignments. Figure 5 shows the direct comparison of the dielectric permittivity *ε*′ vs. BIAS electric field at a given reduced temperature T_I/N_ for all the samples, showing similarities between fully protonated and deuterated 5CB molecules, which substantially differ from partially deuterated 5CB molecules. In both cases, we attribute this behavior to charge distribution and intermolecular interaction symmetry. To deliver more quantitative results for these interactions, high-resolution NMR spectroscopy measurements at high fields for ^1^H and ^2^H atoms are planned.

### 3.5. NMR Relaxometry Study

**Figure 6:**
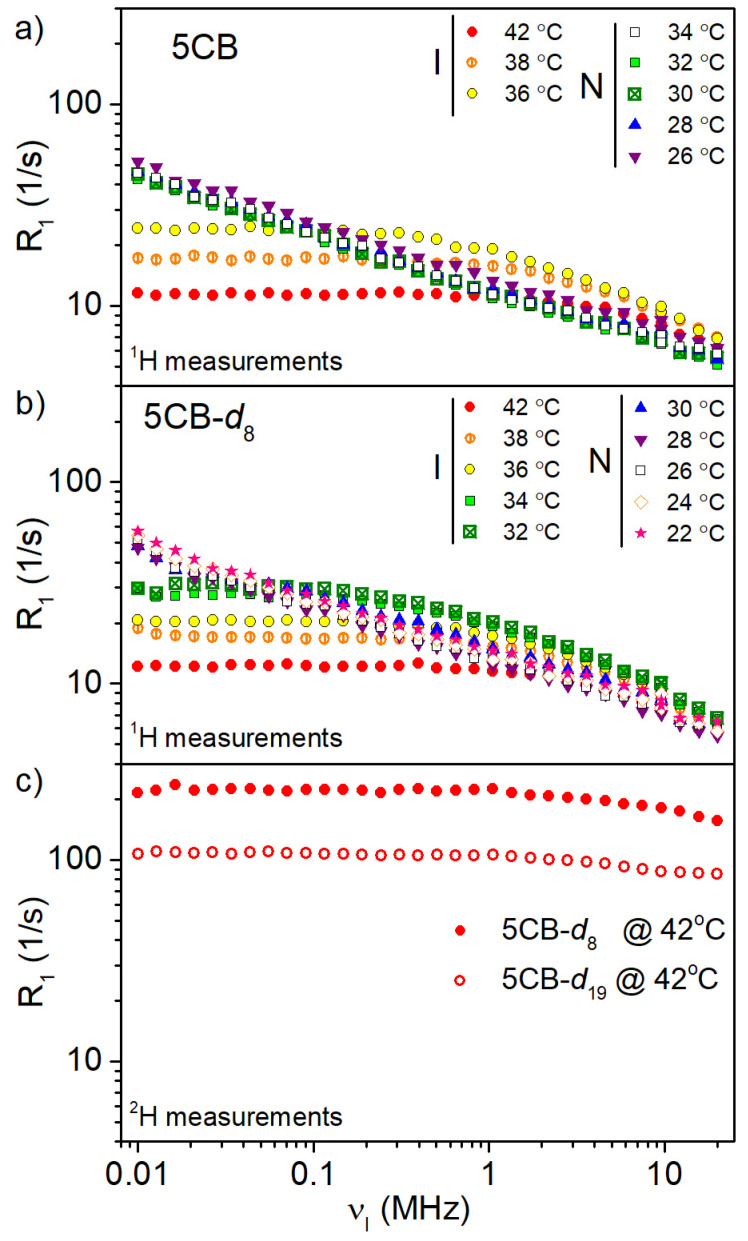
^1^H relaxation dispersion profiles for (**a**) 5CB and (**b**) 5CB as a function of temperature together with ^2^H relaxation dispersion profiles for 5CB-*d*_8_ and 5CB-*d*_19_ in the isotropic phase (**c**).

The spin–lattice relaxation studies of protonated and deuterated samples were performed at different temperatures in isotropic and nematic phases with the use of the fast field cycling nuclear magnetic resonance relaxometry method in the 10 kHz–20 MHz frequency range and at a high field (500 MHz). For the 5CB-*d*_19_ sample, only the isotropic phase was investigated because the dramatic shortening of spin–spin relaxation time prevents us from recording measurable signals in the nematic phase. Figure 6 shows recorded NMRD profiles for investigated samples in isotropic and nematic phases at different temperatures. In the case of the 5CB and 5CB-*d*_8_ samples, the measurements were performed on the ^1^H nucleus in the isotropic and nematic phases. The ^2^H NMRD profiles were recorded for the 5CB-*d*_8_ and 5CB-*d*_19_ samples in the isotropic phase. The too-short T_2_ relaxation times for ^2^H upon crossing to nematic phases did not allow us to use this nucleus to record ^2^H NMRD profiles below the I/N phase transition temperature.

In all studied cases, we observed monoexponential dependence of the magnetization decay or recovery, which proves that the external magnetic field was not large enough to influence the mesogenic molecules’ alignment. A single T_1_ relaxation time was fitted to the experimental data at all studied frequencies. The NMRD profiles were analyzed according to theoretical approaches used for the description of the relaxation mechanisms, as follows:

- Local rotations/reorientations (R) of LC molecule around the molecular axis; the Woessner model with appropriate modifications was used to describe the dynamics in the isotropic and nematic phases.
- Translational self-diffusion (SD) is a type of motion that can be affected by local structure. In the isotropic phase, we used the Torrey model for the isotropic self-diffusion constant. In the nematic phase, the model was extended using the Zumer and Vilfan approach to account for anisotropy of the D constant parallel and perpendicular.
- Order director fluctuations (ODF) describe the orientational fluctuations of nematic director *n* with respect to its time-average orientation. This model reflects the collective reorientational motions of the mesogenic molecules.
- The local order fluctuations (OF) are a contribution that can occur in the isotropic phase just above the N/I temperature, showing square-root power law dependence. Both ODF and OF are collective-type motions that can give similar fits but with different model parameter values.

The general equation used to analyze the NMRD profiles of studied LC systems considers the sum of contributions to relaxation rates R_1_ accounting for particular mesophase structures and types of motions. The contributions are statistically independent from each other or have distinct time scales.

(1) $${\left(\frac{1}{T_{1}}\right)}_{Iso}={\left(\frac{1}{T_{1}}\right)}_{R}+{\left(\frac{1}{T_{1}}\right)}_{SDi}+{\left(\frac{1}{T_{1}}\right)}_{ODF|OF}.$$

We used the following models for the isotropic phase to describe the molecular dynamics. For the spin–lattice relaxation contribution by local rotations/reorientations (*R*) around the long (*z*) and short (*x*) molecular axes, the relaxation rate is given by the Nordio model [34]:

(2) $${\left(\frac{1}{T_{1}}\right)}_{R}=\frac{3}{4}K_{D}\left[J_{R}^{\left(1\right)}(\omega_{L})+J_{R}^{\left(2\right)}(2\omega_{L})\right],$$

where $\omega_{L}=2\pi \nu_{L}$ is the Larmor frequency, and the spectral density function, *J_R_*^(*k*)^ (for *k* = 1, 2), is written as:

(3) $$J_{R}^{\left(k\right)}=\frac{4}{3}{\left(k\right)}^{2}\sum_{m=0}^{2}\frac{\bar{{\left|d_{m,0}^{2}\left(\alpha_{ij}\right)\right|}^{2}}}{r_{ij}^{6}}\times c\left(k,\;m\right)\frac{{\left(\tau_{k,\;m}^{2}\right)}^{-1}}{k^{2}\omega_{0}^{2}+{\left(\tau_{k,\;m}^{2}\right)}^{-2}}$$

where *τ_k_*_,*m*_^2^ depends on correlation times for rotations around a short (*τ_x_*) and long molecular axis (*τ_z_*):

(4) $${\left(\tau_{k,\;m}^{2}\right)}^{-1}=\tau_{z}\left[\frac{1}{\beta_{k,m}^{2}}+\left(\frac{\tau_{x}}{\tau_{z}}-1\right)m^{2}\right].$$

In Equations (3) and (4), *c*(*k*, *m*) and *β_k_*_,*m*_^2^ are numerical functions, and $d_{m,0}^{2}\left(\alpha_{ij}\right)$ are the reduced Winger matrices [35].

Torrey’s model, associated with the translational self-diffusion for isotropic-like systems, can be written in the form [36,37]

(5) $${\left(\frac{1}{T_{1}}\right)}_{{SD}_{i}}=\frac{9}{8}\gamma^{4}\hbar^{2}{\left(\frac{\mu_{0}}{4\pi }\right)}^{2}\frac{n\tau_{D}}{d^{3}}\times \left[T\left(\alpha ,\omega \tau_{D}\right)+4T\left(\alpha ,2\omega \tau_{D}\right)\right],$$

where *d* is the closest distance between the molecules, $\tau_{D}$ is the average time between molecular translational jumps *n*–^1^H spin density, $\alpha =\langle a^{2}\rangle /12d^{2}$, and $\langle a^{2}\rangle =6\tau_{D}D_{i}$ is the mean square root of the molecular jump distance, *D_i_* is the diffusion constant, and $T\left(\alpha ,x\right)$ are analytical functions [36,37].

The OF relaxation contribution can be described by the following expression [37]

(6) $${\left(\frac{1}{T_{1}}\right)}_{OF}=\frac{A_{OF}}{\omega_{L}^{1/2}}\int_{0}^{\nu_{cM}^{OPF}/\nu_{L}}\frac{\sqrt{x}}{1+{\left(x+\nu_{0}^{OF}/\nu_{L}\right)}^{2}}dx,$$

where *A_OF_*, $\nu_{cM}^{OF}$, and $\nu_{0}^{OF}$ depend on viscoelastic parameters in the isotropic phase and denote the strength of the OF process at the high and low cutoff frequency.

In the isotropic phase for 5CB and 5CB-*d*_8_ liquid crystal, the ^1^H and ^2^H NMRD profiles show similar shapes. In the low-frequency region, a plateau was observed. The R_1_ values at this plateau vary with temperature from 11.5 to 24 s^−1^ in 5CB and 12 to 30 s^−1^ in 5CB-*d*_8_, by cooling from 42 to 36 °C and 42 to 32 °C, respectively.

To describe the intermolecular interactions and motions in the nematic phase, we used the same general approach given in Equation (1) but with appropriate modifications for the given molecular processes. In the case of molecular rotations/reorientations along the molecular axis, we used the same Nordio model as in the case of the isotropic phase. For the description of translational self-diffusion (SD) processes, we used the Torrey model expanded by Žumer and Vilfan, who took into account the anisotropy of the system. As a result, the following expression for the spin–lattice relaxation contribution related to self-diffusion was considered [38]:

(7) $${\left(\frac{1}{T_{1}}\right)}_{{SD}_{a}}=\frac{9}{8}\gamma^{4}\hbar^{2}\frac{n\tau_{D}}{d^{3}}\times Q\left(\omega_{L}\tau_{D},\frac{\langle a_{⊥}^{2}\rangle }{d^{2}},\;\frac{l}{d},\frac{D_{∥}}{D_{⊥}}\right),$$

where $\langle a_{⊥}^{2}\rangle =4\tau_{D_{⊥}}D_{⊥}$ is the width of the molecule, *l* is the layer thickness, *n* is the spin density, $\tau_{D_{⊥}}$ is the mean square of molecular jump time, and *D*_⊥_ and *D*_∥_ are diffusion constants of molecules in two perpendicular directions to the nematic director; *Q* is a dimensionless function calculated numerically. In the nematic phase, we also used the model for order director fluctuations (ODF) instead of local order fluctuations as in the I phase.

(8) $${\left(\frac{1}{T_{1}}\right)}_{ODF}=\frac{A_{ODF}}{\omega^{1/2}}\left[f\left(\frac{\omega_{ODFmax}}{\omega }\right)-f\left(\frac{\omega_{ODFmin}}{\omega }\right)\right]$$

where *f*(*x*) is the cutoff function and the prefactor:

(9) $$A_{ODF}=\left(9/8\right){\left(\mu_{0}/4\pi \right)}^{2}\gamma^{4}\hbar^{2}k_{B}TS^{2}\eta^{1/2}/\left(\sqrt{2}\pi K^{3/2}a_{eff}^{6}\right)$$

where $a_{eff}^{6}$ depends on the interproton distances and interproton vector angles with respect to the long molecular axis. *η* and *K* are the viscosity and elastic constant of nematic liquid crystal.

**Figure 7:**
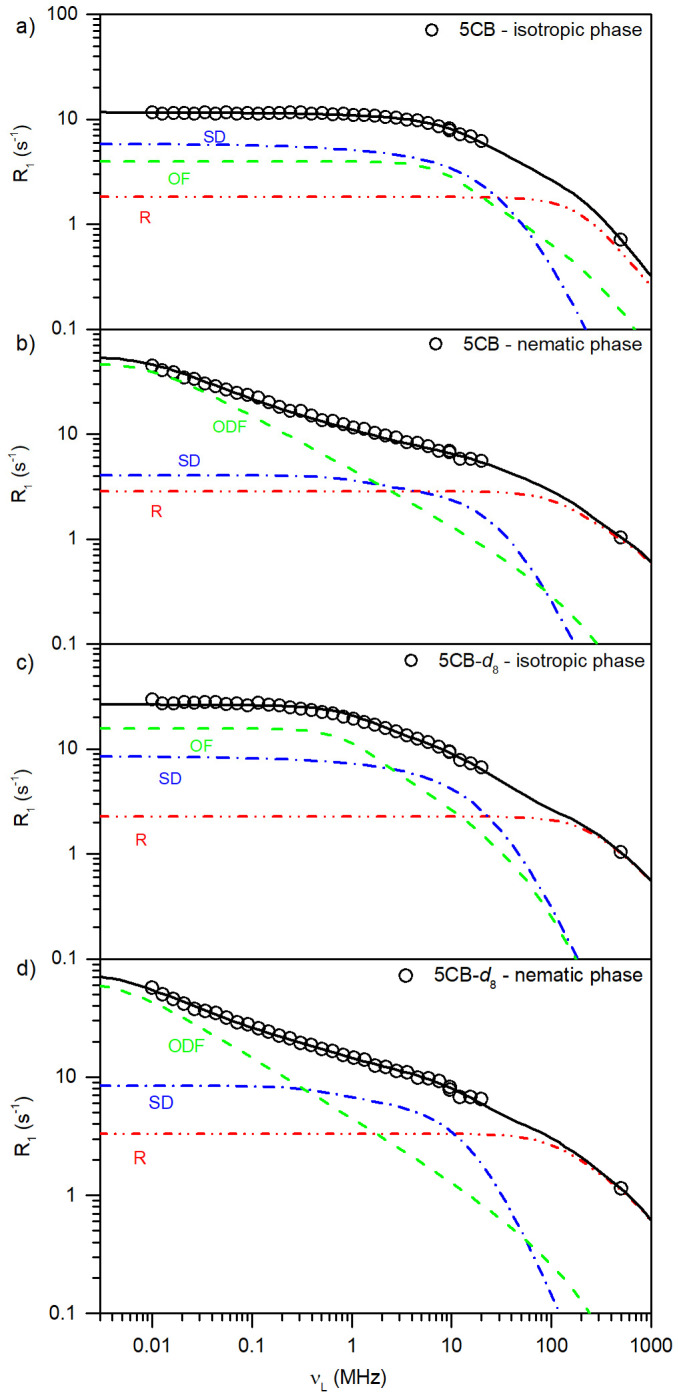
^1^H and ^2^H experimental NMRD profiles R_1_ for 5CB in isotropic phase (**a**) and nematic phase (**b**) and for 5CB-*d*_8_ in isotropic phase (**c**) and nematic phase (**d**), together with the best fits obtained with the model given by Equation (1).

The NMRD profiles measured in the nematic phase change significantly as to the isotropic phase, showing strong dispersion dependence in the low-frequency range (Figure 5). Figure 7 shows the best fits of the mentioned models to experimental data for 5CB at 42 °C and 5CB-*d*_8_ at 34 °C in the isotropic phase and at 30 °C and 22 °C in the nematic phase, respectively. The experimental data also included R_1_ values measured at 500 MHz, which were of great importance in the analysis of the results because they allowed for a more faithful reproduction of the shape of the relaxation profiles. The fitting parameters are gathered in Table 4 and Table 5.

The results show that the fully protonated 5CB liquid crystal is characterized by smaller order fluctuations and a faster self-diffusion coefficient compared to 5CB-*d*_8_. These results stay in agreement with conclusions obtained from other methods for studied systems, reflecting the nonuniform alignment of 5CB-*d*_8_ molecules close to T_I/N_ and additional phase transitions in the partially deuterated sample, smaller mass of the molecules, and weaker intermolecular interactions.

**Figure 8:**
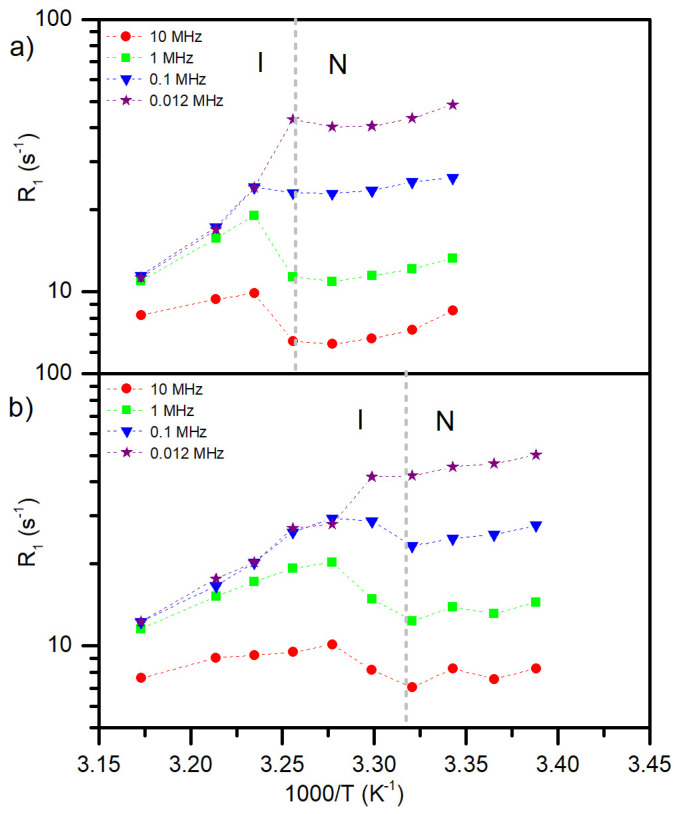
Temperature dependence of R_1_ at chosen ^1^H Larmor frequencies. The vertical dashed lines correspond to the phase transition determined by DSC on cooling [39] for 5CB (**a**) and 5CB-d_8_ (**b**).

Figure 8 shows the dependence of the relaxation rate R_1_ in different phases for selected Larmor frequencies. The dashed gray line indicates the I/N phase transition temperature determined from DSC measurement upon cooling. Within the isotropic phase, the R_1_ values increase with a decrease in the temperature at all Larmor frequencies for the 5CB and 5CB-*d*_8_ samples. At the temperature of the I/N phase transition and further, a change in the R_1_ dependence is observed. For lower frequencies (≤100 kHz), an abrupt slope change is observed at the I/N transition, followed by almost constant R_1_ throughout the N phase for 5CB, whereas an increase in R_1_ value is observed for 5CB-*d*_8_. Moreover, in the case of the 5CB-*d*_8_ sample below the I/N phase transition temperature, the NMRD profile recorded at 100 kHz frequency shows a kind of curvature that is not seen for 5CB. This can originate from the pretransition effect when the system prepares to create the I/N domain structure, evidenced by the POM texture images for the 5CB-*d*_8_ sample before reaching the uniform nematic phase. It is worth noticing that DSC measurements did not detect this state as it is not related to the change in the heat flow in the sample (or the change is too small to be detected in the applied experimental conditions) but with the change of molecular alignment and local self-organization.

## 4. Conclusions

In this work, we investigated the influence of selective deuteration of 5CB liquid crystals on molecular dynamics, phase transitions, textures, and dielectric properties. Deuteration plays an important role in chemical modifications of liquid crystal materials utilized in near-infrared applications to reduce common disadvantages, like parasitic absorption of LCs. Our studies have shown that a fully deuterated 5CB sample exhibits the same phase transition sequence as fully protonated material with almost the same range of nematic phase occurrence shifted only by 3 °C lower compared to 5CB. The protonated 5CB molecules exhibit a sharp transition from isotropic to nematic phase with uniform alignment of the molecules. In the 5CB-*d*_19_ sample, we detected a nematic/isotropic domain structure at the I/N transition, which transforms into a homogenous nematic phase with uniform molecule alignment within 0.3 °C. Moreover, the dielectric properties and the dependence of the dielectric permittivity in the function of the applied electric field and the threshold field are very similar to those of 5CB. Significant differences in properties were detected in partially deuterated 5CB molecules (5CB-*d*_8_). The thermal analysis has shown a similar sequence of thermal events during the heating stage but with big differences in individual temperatures of the phase transitions. During the cooling of the 5CB-*d8* sample, the differences in phase transition temperature were also significant. Additionally, the sample showed no partial crystallization in the nematic phase before the glass transition temperature. The characteristic I/N phase transition temperature is shifted by 10 °C lower as protonated 5CB molecules and 6 °C lower as fully deuterated 5CB-*d*_19_ molecules. The texture analysis showed a domain structure that spans over 2 °C with no uniform alignment of the molecules. Moreover, the dependence of the dielectric permittivity in the function of the external electric field differs significantly for both fully protonated and deuterated 5CB samples. The FFC NMR measurements performed indicate the high sensitivity of this method, especially at low Larmor frequencies, for the detection of pretransitional effects and its influence on the molecular dynamics in the investigated samples, not seen by other commonly used experimental techniques for the study of liquid crystals. In conclusion, we state that deuteration can be a suitable method for optimizing the LC properties for utilization in NIR applications, but the process has to be performed thoroughly. Partial deuteration introduces changes in the phase transition sequence, alters dielectric properties and molecular dynamics, and causes the appearance of the domain structure in the nematic phase close to the I/N transition. The possible origin of such behavior is in the change in the symmetry of charge distribution, mass, and intermolecular interaction in partially deuterated systems.

## Figures and Tables

**Table 1:** Acronyms, chemical structures, chemical and isotopic purities of the studied materials.

Acronym	Chemical Structure	Chemical Purity	Isotopic Purity
5CB	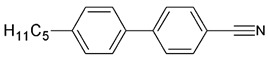	99.8%	^1^H—100%
5CB*-d*_8_	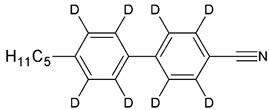	99.4%	^2^H total—47% ^2^H rings—96% ^2^H chain—11%
5CB*-d*_19_	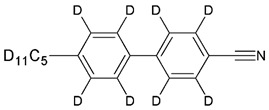	99.5%	^2^H total—96% ^2^H rings—93% ^2^H chain—97%

Chemical purity determined by gas chromatography–mass spectrometry method (GC-MS); isotopic purity determined by ^1^H nuclear magnetic resonance method (NMR).

**Table 2:** The characteristic phase transition temperatures obtained with the DSC and POM method.

Sample	Phase Transition Temperature/°C from DSC								T_I/N_/°C from POM
	Heating				Cooling				Cooling
	Glass Tr	Cold Cr	Cr/N	N/I	I/N	Partial Cr	Glass Tr	Cr	
5CB	−68.1	−29.5	23.7	34.8	32.7	−28.4	−69.1	--	34.2
5CB-*d*_8_	−67.1	−18.6	18.4	27.7	25.0	--	−67.5	−115.2	27.6–25.8
5CB-*d*_19_	−69.3	−26.6	21.5	32.5	30.7	−18.9	−69.1	−103.5	32.0–31.7

Glass Tr—glass transition; Cold Cr—cold crystallization, Cr/N—crystal-to-nematic phase transition; N/I—nematic-to-isotropic phase transition; I/N—isotropic-to-nematic phase transition; Partial Cr—partial crystallization; Cr—crystallization.

**Table 3:** Values of Δ*ε* and U_th_ for 5CB, 5CB-*d*_8_, and 5CB-*d*_19_ at different temperatures in the nematic phase.

**Sample**	**Δ*ε* (1.1 kHz, T_I/N_-5 °C)**	**U_th_ (1.1 kHz, T_I/N_-5 °C)**
5CB	10.5	4.4
5CB-*d*_8_	9.1	6.6
5CB-*d*_19_	10.1	3.9
	**Δε (1.1 kHz, T_I/N_-10 °C)**	**U_th_ (1.1 kHz, T_I/N_-10 °C)**
5CB	11.9	4.4
5CB-*d*_8_	10.7	6.5
5CB-*d*_19_	11.4	4
	**Δ*ε* (1.1 kHz, T_I/N_-15 °C)**	**U_th_ (1.1 kHz, T_I/N_-15 °C)**
5CB	12.8	4.4
5CB-*d*_8_	11.7	6.9
5CB-*d*_19_	12.2	4.3
	**Δ*ε* (1.1 kHz, T_I/N_-20 °C)**	**U_th_ (1.1 kHz, T_I/N_-20 °C)**
5CB	13.3	4.4
5CB-*d*_8_	12.5	6.9
5CB-*d*_19_	12.8	5

**Table 4:** The isotropic phase, where the order parameter S_0_ = 0, low cutoff frequency ω_cmin_/2π = 0 Hz, the geometrical factors A_0_ = 6 × 10^57^ m^−6^, A_1_ = 3 × 10^57^ m^−6^, A_2_ = 9 × 10^57^ m^−6^, the distance of the closest approach d = 5 × 10^−10^ m, and n = 4.6 × 10^22^.

Fitting Parameter	5CB, 42 °C	5CB-*d*_8_, 34 °C	1/T_1_ Model
*τ_z_*/s	3.30 × 10^−10^	4.67 × 10^−9^	R
*τ_x_*/s	5.02 × 10^−8^	5.48 × 10^−8^	R
*D*/m^2^s^−1^	1.59 × 10^−11^	1.08 × 10^−11^	SDi
*A_OF_*/s^−3/2^	1.04 × 10^4^	1.30 × 10^4^	OF
$\nu_{0}^{OF}$/Hz	4.59 × 10^5^	1.59 × 10^4^	OF
$\nu_{cM}^{OF}$/Hz	9.00 × 10^8^	1.00 × 10^8^	OF

**Table 5:** The nematic phase, where the order parameter S_0_ = 5 and the geometrical factors A_0_, A_1_, A_2_, d, and n take the same values as in the isotropic phase.

Fitting Parameter	5CB, 30 °C	5CB-*d*_8_, 22 °C	1/T_1_ Model
*τ_z_*/s	4.05 × 10^−9^	3.14 × 10^−9^	R
*τ_x_*/s	1.84 × 10^−8^	1.68 × 10^−8^	R
*τ_D_*/s	3.09 × 10^−9^	6.43 × 10^−9^	SDa
*A_ODF_*/s^−3/2^	4.78 × 10^3^	4.69 × 10^3^	ODF
$\nu_{cm}^{OF}$/Hz	8.00 × 10^3^	4.31 × 10^3^	ODF
$\nu_{cM}^{OF}$/Hz	5.00 × 10^8^	3.88 × 10^8^	ODF

## Data Availability

The raw data supporting the conclusions of this article will be made available by the authors on request.
